# First insights of peptidoglycan amidation in Gram-positive bacteria - the high-resolution crystal structure of *Staphylococcus aureus* glutamine amidotransferase GatD

**DOI:** 10.1038/s41598-018-22986-3

**Published:** 2018-03-28

**Authors:** Francisco Leisico, Diana V. Vieira, Teresa A. Figueiredo, Micael Silva, Eurico J. Cabrita, Rita G. Sobral, Ana Madalena Ludovice, José Trincão, Maria João Romão, Hermínia de Lencastre, Teresa Santos-Silva

**Affiliations:** 1 UCIBIO, Departamento de Química, Faculdade de Ciências e Tecnologia, Universidade Nova de Lisboa, Caparica, Portugal; 2 Oxford Protein Production Facility, Research Complex at Harwell, Didcot, United Kingdom; 3 UCIBIO, Departamento de Ciências da Vida, Faculdade de Ciências e Tecnologia, Universidade Nova de Lisboa, Caparica, Portugal; 4 Laboratory of Molecular Genetics, Microbiology of Human Pathogens Unit, Instituto de Tecnologia Química e Biológica António Xavier da Universidade Nova de Lisboa, Oeiras, Portugal; 5 Diamond Light Source, Didcot, United Kingdom; 6 Laboratory of Microbiology and Infectious Diseases, The Rockefeller University, New York, USA

## Abstract

Gram-positive bacteria homeostasis and antibiotic resistance mechanisms are dependent on the intricate architecture of the cell wall, where amidated peptidoglycan plays an important role. The amidation reaction is carried out by the bi-enzymatic complex MurT-GatD, for which biochemical and structural information is very scarce. In this work, we report the first crystal structure of the glutamine amidotransferase member of this complex, GatD from *Staphylococcus aureus*, at 1.85 Å resolution. A glutamine molecule is found close to the active site funnel, hydrogen-bonded to the conserved R128. *In vitro* functional studies using ^1^H-NMR spectroscopy showed that *S. aureus* MurT-GatD complex has glutaminase activity even in the absence of lipid II, the MurT substrate. In addition, we produced R128A, C94A and H189A mutants, which were totally inactive for glutamine deamidation, revealing their essential role in substrate sequestration and catalytic reaction. GatD from *S. aureus* and other pathogenic bacteria share high identity to enzymes involved in cobalamin biosynthesis, which can be grouped in a new sub-family of glutamine amidotransferases. Given the ubiquitous presence of GatD, these results provide significant insights into the molecular basis of the so far undisclosed amidation mechanism, contributing to the development of alternative therapeutics to fight infections.

## Introduction

*Staphylococcus aureus* is considered one of the most important human Gram-positive bacterial pathogens due to its capacity to acquire and develop antibiotic resistance, causing high levels of mortality both in hospital and community settings^1^. Methicillin-resistant *S. aureus* (MRSA) are resistant to all ß-lactam antibiotics and show higher propensity to accumulate resistance to other classes of antibiotics. Fighting *S. aureus* is an urgent task that requires finding new drugs for new targets^2^. In this quest, peptidoglycan (PG) biosynthesis plays an important role since PG is essential for bacterial survival, cell shape maintenance and turgor pressure counterbalance^3^.

PG biosynthesis is a complex process that takes place in several sequential enzymatic steps^2^ (Supplementary Fig. S1). The first step of PG biosynthesis occurs in the cytoplasm, where the nucleotide carbohydrate UDP-MurNAc (UDP-N-acetyl muramic acid) is covalently linked to a small pentapeptide (L-alanine-D-iso-glutamate-L-lysine-D-alanine-D-alanine) forming UDP-MurNAc-pentapeptide. At the membrane level, an undecaprenyl-phosphate lipid carrier binds to the precursor molecule, generating lipid I (Step 1 in Supplementary Fig. S1). Subsequently, a GlcNAc unit is transferred from UDP-GlcNAc to the MurNAc carbohydrate of lipid I, leading to the formation of lipid II (Step 2 in Supplementary Fig. S1). This structure suffers several modifications, such as the addition of a pentaglycine bridge at the L-lysine residue of the pentapeptide, the O-acetylation of MurNAc carbohydrate and the amidation of the D-iso-glutamate residue into D-iso-glutamine (Step 3 in Supplementary Fig. S1). Finally, the PG monomer is translocated across the cytoplasmic membrane and assembled into the growing PG, through transglycosylation and transpeptidation reactions^4^ (Step 4 in Supplementary Fig. S1).

The PG of *S. aureus* is characterized by a high degree of crosslinking and almost complete lack of carboxyl groups, due to the amidation of the D-iso-glutamate residue of the stem peptide^5^. This amidation of PG structure has a major impact in β-lactam antibiotic resistance, as shown with the *femC* (*glnRA*) mutant of MRSA^6^. In vancomycin-resistant *S. aureus* strains, it was recently shown that the presence of D-iso-glutamine in lipid II stem peptide enhances the binding affinity to chloroeremomycin and oritavancin^7^.

**Figure 1:**
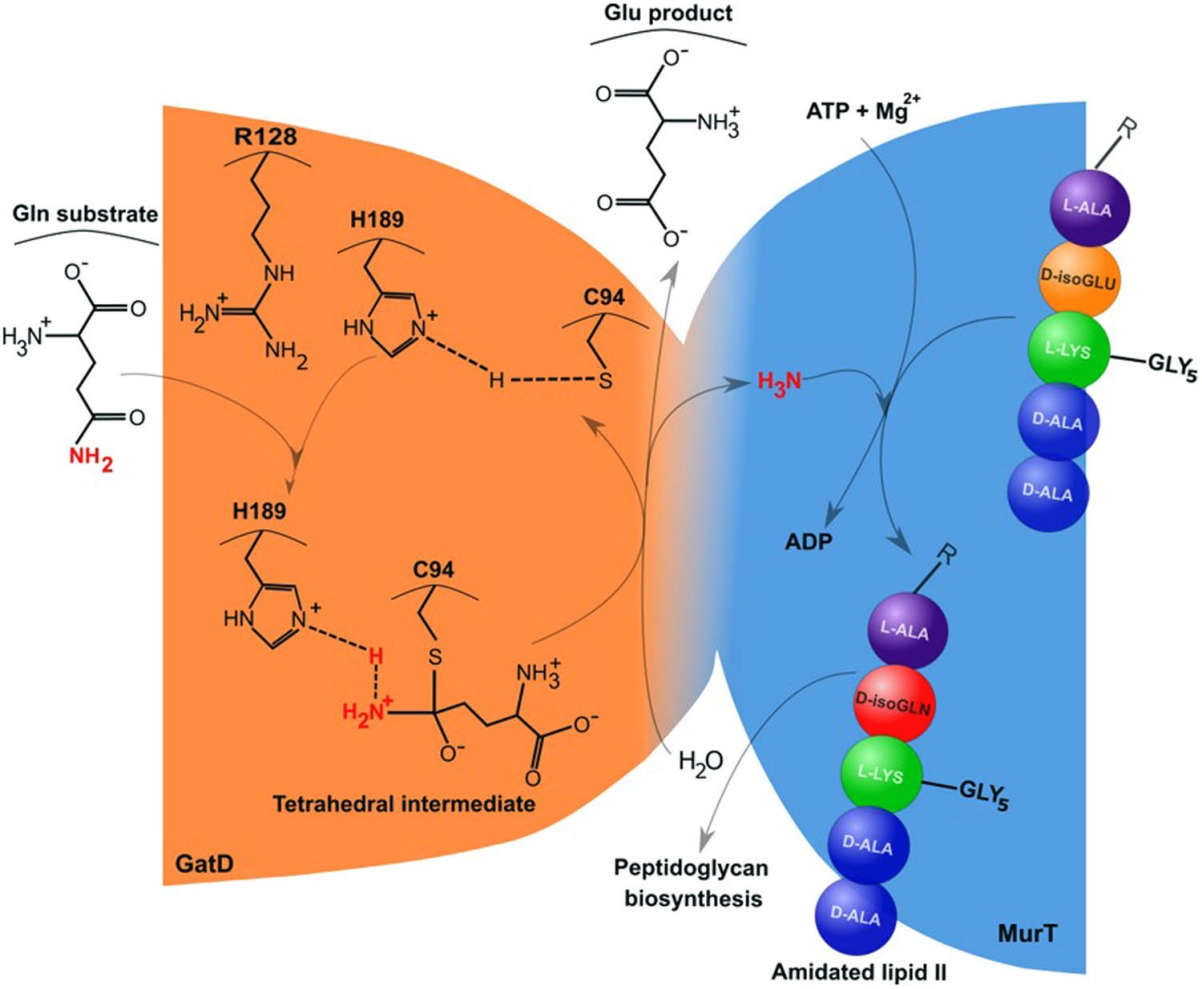
Schematic representation of lipid II amidation in *S. aureus*. The MurT-GatD-catalysed amidation reaction in the PG biosynthetic pathway is divided in two stages: first, in GatD, deamidation of glutamine releases ammonia, which travels to MurT, where it is used to amidate the stem peptide residue D-iso-Glu of lipid II to D-iso-Gln in the amidated lipid II.

Recently, MurT and GatD enzymes were identified as responsible for catalyzing glutamate amidation in *S. aureus* PG stem peptides (Fig. 1)^8,9^. The *murT* and *gatD* gene arrangement found in *S. aureus* is conserved among Gram-positive bacteria^8,9^. Genetic studies showed that the impairment of the MurT-GatD complex reduces the bacterial growth rate, resistance to β-lactam antibiotics and to lysozyme^9,10^. Moreover, *in vitro* experiments showed that the MurT-GatD complex catalyzes glutamate amidation of lipid II using L-glutamine as a direct amine donor, when ATP and Mg^2+^ were present. Maximum lipid II amidation was achieved with a MurT:GatD molar ratio of 1:1, suggesting the formation of a heteromeric complex. Site-directed mutagenesis identified GatD C94 as an important residue for catalysis^8^. Similarly, Zapun and co-workers showed that lipid II amidation in *Streptococcus pneumoniae* strain R6 is catalyzed by MurT-GatD complex, which is essential for PG cross-linking and for cell viability^11^.

MurT belongs to the family of Mur ligases, which are cytoplasmic enzymes responsible for the sequential addition of amino acid residues to the growing UDP-MurNAc precursor, containing the conserved motifs for ATP and Mg^2+^ binding. GatD shows high sequence homology to the glutaminase domain of glutamine amidotransferase (GATase) enzymes, in particular to cobyric acid synthase, which is involved in cobalamin biosynthesis^9^.

The amidation reactions are catalyzed by different domains of a single polypeptide chain or by different subunits of a protein complex and it can be divided into two steps. The first step is the glutaminase reaction, in which glutamine is converted into ammonia and glutamate. The NH_3_ molecule is then transferred to the synthase domain/subunit through a solvent channel that prevents its protonation to ammonium. Finally, ammonia is added to a specific substrate, in a synthase reaction. Glutamine is used as an amide donor in several pathways that use amino acids, carbohydrates, nucleotides, co-enzymes and antibiotics as substrates^12^.

Based on amino acid sequence analysis, the glutaminase domain in GATases have been divided in two classes, with distinct protein folds and very different active sites. The enzymes that belong to class I have a conserved catalytic triad at the active site, formed by a cysteine, a histidine and a glutamate residue, and are usually called triad GATases. Structurally characterized triad GATases include: anthranilate synthase (AS), carbamoyl phosphate synthetase (CPS), cytidine triphosphate synthetase, formylglycinamidine ribonucleotide amidotransferase (FGAR-AT), guanosine monophosphate synthetase, imidazole glycerol phosphate synthase (IGPS) and pyridoxal 5′-phosphate synthase (PLPS)^13^. In class II enzymes, the main characteristics are the position of the essential catalytic cysteine at the N-terminal and no recognizable catalytic triad in the sequence^12,14^. Asparagine synthetase B, glucosamine-6-phosphate synthase, glutamate synthase and glutamine phosphoribosylpyrophosphate amidotransferase are examples of structurally characterized class II GATases^13^.

A reaction mechanism has been proposed for class I triad GATases, based on structural evidences^13^. Generally, the glutamate residue is hydrogen-bonded to the side chain of the catalytic histidine, restricting its rotation and inducing its polarization. The histidine imidazole ring can then deprotonate the nucleophilic cysteine, increasing its reactivity. The catalytic cysteine, in the form of a thiolate, attacks the carbonyl carbon atom of the GATase substrate, the glutamine carboxamide group, forming a tetrahedral intermediate^15,16^. The stabilization of this negatively charged intermediate is achieved through hydrogen bonds with main chain atoms at the conserved “oxyanion hole”. After the protonation of the amino group in the tetrahedral intermediate, an ammonia molecule is released via a channel to the synthase domain. Finally, the former glutamine/new glutamate is released from the glutamyl-thioester via an acid/base catalysis of a nucleophile attacking water^12^.

In this work, we combined X-ray crystallography and Nuclear Magnetic Resonance (NMR) spectroscopy to study the glutaminase activity of *S. aureus* strain COL GatD. The crystal structure was solved at 1.85 Å resolution, unraveling singular features of GATase architecture and important residues at the active site. Site-directed mutagenesis revealed the key amino acids involved in MurT-GatD glutaminase activity and substrate sequestration.

## Results/Discussion

### Overall structure

**Figure 2:**
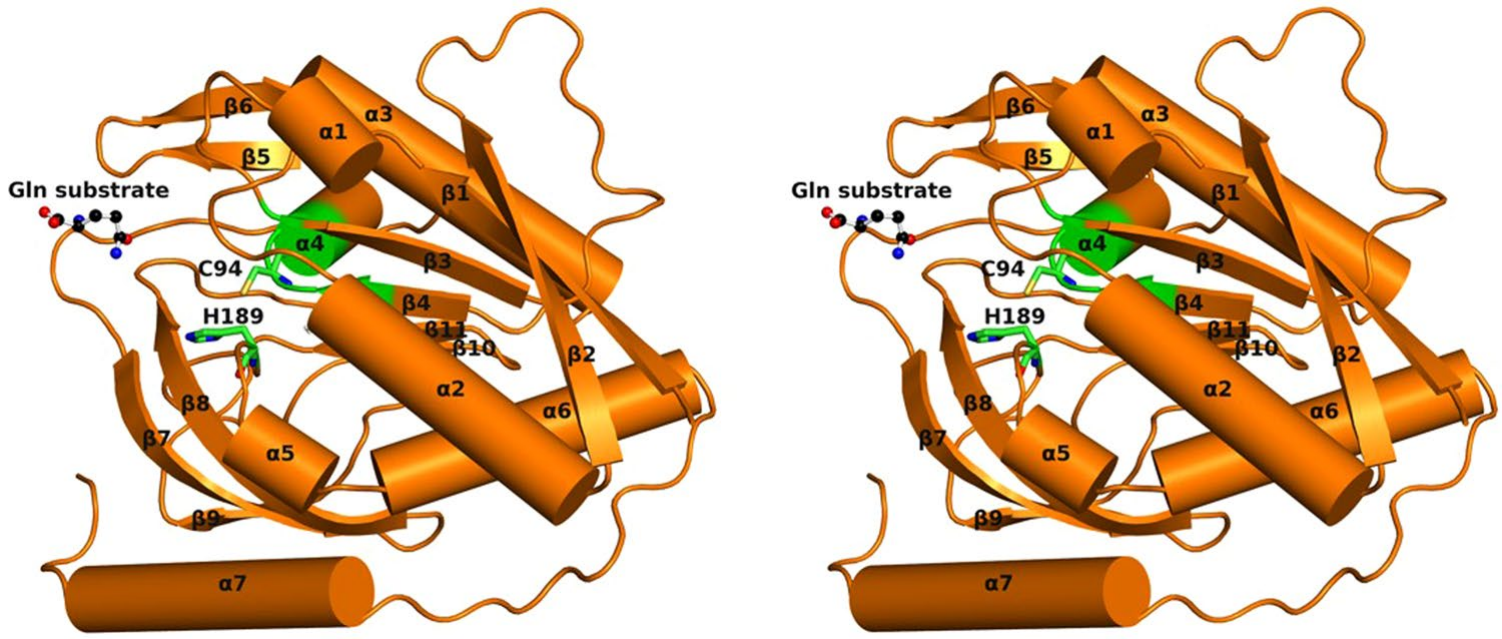
Ribbon representation of *S. aureus* GatD 3D structure. GatD chain A with secondary structural elements labelled. The active site residues (C94 and H189) are represented as sticks with carbon atoms colored green and free glutamine as ball and stick with carbon atoms colored black. The structure is shown in wall-eyed stereo view.

GatD crystallized in P2_1_2_1_2_1_ space group, with 2 molecules in the asymmetric unit, forming a non-physiological dimer via non-crystallographic symmetry operations (validated by visual inspection and using PISA software^17^). Molecule A (Fig. 2) corresponds to the full-length GatD protein with 243 amino acids, while molecule B lacks electron density for the last four residues of the polypeptide chain. A free glutamine molecule was found at the surface of GatD chain A. The GatD molecule has a globular shape with the approximate dimensions 50 × 54 × 33 Å^3^.

**Figure 3:**
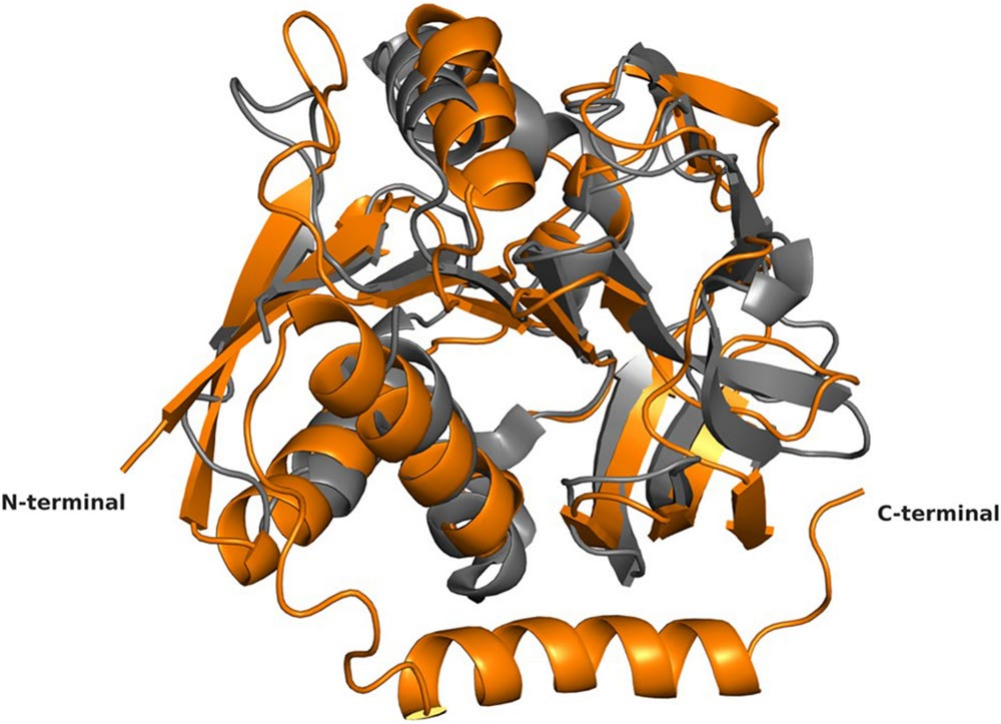
3D structural alignment of *S. aureus* GatD and GATase domain of imidazole glycerol phosphate synthase. Superposition of Cα-atoms of GatD chain A and IGPS from *T. maritima* (PDB ID 3ZR4) chain B. Both structures are represented as ribbons with GatD colored orange and IGPS dark grey.

The enzyme adopts an open α/β structure fold, similar to the glutaminase domain from a wide range of GATases^15^. Indeed, using the GatD model as input in the protein structure comparison service PDBeFold at European Bioinformatics Institute (http://www.ebi.ac.uk/msd-srv/ssm)^18^, several class I triad GATases were identified (Table 1). The most similar structure found is the *Thermotoga maritima* IGPS, with a RMSD of 2.58 Å (162 residues aligned) upon superposition, with an evident overall structure conservation (Fig. 3).

**Table 1:** Similarity of *S. aureus* GatD Structure with PDB Archive (PDBeFold).

Protein	Organism	Biosynthetic pathway	Protein length	Identity^a^	RMSD (Å)	PDB ID:chain	Reference
IGPS	*Thermotoga maritima*	Histidine	201	179 (16%)	2.58	3ZR4:B	^21,43^
PLPS	*Thermotoga maritima*	Vitamin B6	185	163 (18%)	2.61	2ISS:D	^44^
PLPS	*Geobacillus stearothermophilus*	Vitamin B6	202	176 (18%)	2.65	1Q7R:A	^44^
FGAR-AT	*Thermotoga maritima*	Purines	212	168 (16%)	2.67	3D54:D	^45^
PLPS	*Geobacillus kaustophilus* HTA426	Vitamin B6	188	171 (16%)	2.77	4WXY:B	^46^
PLPS	*Bacillus subtilis*	Vitamin B6	187	168 (16%)	2.80	2NV0:B	^47^
PLPS	*Plasmodium berghei*	Vitamin B6	217	179 (16%)	2.99	4ADS:I	^48^
PLPS	*Plasmodium falciparum*	Vitamin B6	216	178 (16%)	2.99	2ABW:A	^49^
AS	*Sulfolobus solfataricus*	Tryptophan	195	160 (14%)	3.13	1QDL:B	^25^
AS	*Serratia marcescens*	Tryptophan	195	193 (16%)	3.07	1I7Q:D	^50^

The 3D alignment was performed in pairwise form using molecule A of *S. aureus* GatD structure. The target structures are ranked by RMSD from the 3D superposition of Cα-atoms. Protein names are abbreviated, as following: imidazole glycerol phosphate synthase, IGPS; piridoxal phosphate synthase, PLPS; formylglycinamidine ribonucleotide amidotransferase, FGAR-AT; anthranilate synthase, AS. Model 1I7Q is here included since it is used for structural comparison of triad GATases active site. ^a^Identity includes the number of aligned residues with *S. aureus* GatD sequence and the percentage of identical residues in parenthesis.

The *S. aureus* GatD crystal structure shows the characteristic β-sheet core composed by six strands (β1, β2, β3, β4, β10 and β11), all parallel except for β10 (Fig. 2). This β-sheet core is flanked on one side by two helices (α3 and α4) and a two-stranded antiparallel β-sheets (β5 and β6), similar to what has been described for other GATase structures^19^. The other side of the β-sheet core is covered by a three-stranded antiparallel β-sheets (β7, β8 and β9) and by four α-helices (α1, α2, α5 and α6) (Fig. 2). Interestingly, GatD holds an extra 17 residues long α-helix (α7) at the C-terminal that is absent in all GATases characterized so far^19^ (Fig. 3 and Supplementary Fig. S2). This α-helix is located at the surface of the protein, as depicted in Fig. 2.

The 3D models of bi-enzymatic complexes selected for structural comparison show a common region for the interaction between the GATase and the corresponding synthase domain. Upon protein-protein interaction, conformational changes occur in the GATase domain that allow the substrate to reach the active site and the catalytic reaction to occur with formation of the ammonia shuttle^13^. The *S. aureus* GatD structure here reported was crystallized without its synthase domain (MurT), consequently, in an unproductive enzymatic form.

### Active site

**Figure 4:**
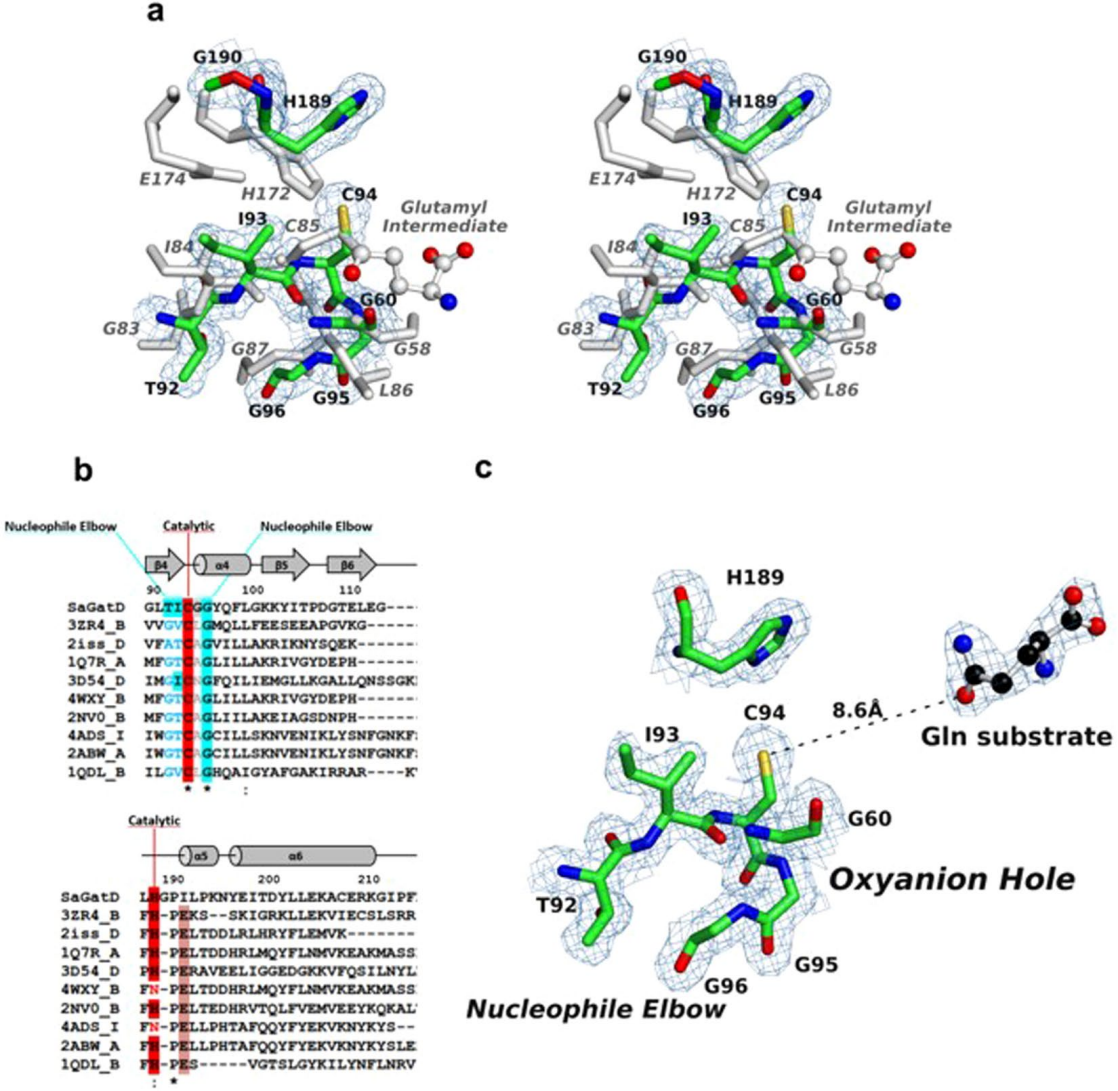
Representation of *S. aureus* GatD active site. (**a**) Structural comparison of the catalytic sites of *S. aureus* GatD (in green) and Anthranilate Synthase (AS) from *S. marcescens* (in grey). The AS crystal structure (PDB ID 1I7Q) includes the glutamyl-thioester intermediate with C85. In AS, the G58 and L86 form the oxyanion hole; the catalytic triad is formed by the C85, H172 and E174; and the G83, I84 and G87 belong to the nucleophile elbow. The superposition is shown in wall-eyed stereo view and the 2Fo-Fc map is contoured at 1.0 σ. (**b**) Partial sequence alignment (using the ClustalW algorithm) of GatD and PDBeFold hits presented in Table 2 showing the sequence conservation of class I GATases active site, apart from the catalytic glutamate. The secondary structural elements from the GatD 3D structure are shown above the aligned sequences, with α-helices represented as cylinders and β-sheets as arrows. (**c**) GatD active site architecture, with the catalytic residues (C94 and H189), the nucleophile elbow and the oxyanion hole residues represented as sticks with carbon atoms colored green. The free glutamine molecule is represented as ball and stick with carbon atoms colored black. The 2Fo-Fc electron density map is contoured at 1.0 σ.

As expected, the active site of *S. aureus* GatD shows high structural similarity to the triad GATases previously mentioned. For comparison, the model of AS from *Serratia marcescens* (PDB ID 1I7Q) was selected since the glutamyl-thioester intermediate was captured in this crystal structure, highlighting the important residues for the reaction. The superposition (RMSD of 3.07 Å, 148 residues aligned) shows only slight differences in the locations of the catalytic residues between both models (Fig. 4a), probably due to conformational changes that occur upon substrate binding, in the presence of the synthase domain.

An important difference between the *S. aureus* GatD and other GATases so far characterized, is the absence of the glutamate residue that usually completes the catalytic triad (E174 in *S. marcescens* AS protein, Fig. 4a). In the structure here reported, glutamate is replaced by a glycine (G190), thereby eliminating any possible polarization effect upon the catalytic histidine H189. In fact, the catalytic role of the conserved glutamate in other GATases is not well understood. In CPS from *Escherichia coli*, site directed mutagenesis analysis revealed that the catalytic cysteine (C269) and histidine (H353) residues are essential for catalysis, but not the conserved glutamate (E355)^20^. Additionally, Hart, E.J., *et al*. showed that the structurally conserved catalytic triad of CPS can act as a functional dyad, since substitution of the neighboring residues of the catalytic histidine (Q310, N311, D334 and Q351) by alanine had no effect on the reaction^15^. IGPS from *T. maritima* is another example of a triad GATase, where biochemical data supported the non-catalytic role of the conserved glutamate^21^. All these evidences point towards the hypothesis that glutamine deamidation can be carried out by cysteine/histidine dyads, which is in agreement with the GatD crystal structure here reported. However, we cannot exclude the hypothesis that GatD residues at the active site may rearrange upon MurT-GatD complex formation (*in vitro* and/or *in vivo*), bringing another acidic residue into play and generating a catalytic triad. The glutamate conservation in class I triad GATase and its absence in *S. aureus* GatD could be related to the specific function of MurT-GatD in the context of peptidoglycan synthesis pathway and should be further studied.

Additional features of GATases active sites, such as the oxyanion hole and the nucleophile elbow, affect glutamine deamidation, empowering the hydrolysis reaction. The oxyanion hole is formed by the amino acid residue next to the catalytic cysteine, commonly a leucine, and by a second residue adjacent to the so-called oxyanion strand^13^ (G58 in *S. marcescens* AS protein, Fig. 4a). The backbone NHs of these residues are oriented in such a way that enable two hydrogen bond interactions with a single oxygen atom from the substrate. In *S. aureus* GatD the residues G95 and G60 of strand β3 are structurally aligned with *S. marcescens* AS, forming the oxyanion hole (Fig. 4a).

Generally, in triad GATases, the catalytic cysteine is located at the edge of a central strand of the β-sheet core and adjacent to an α-helix, forming a “nucleophile elbow” structural motif. The sequence of this motif is conserved among triad GATases and corresponds to G-X-C-X-G^22^ (Supplementary Fig. S2). The nucleophile elbow is flanked by the loop containing the catalytic histidine and glutamate, and by the oxyanion strand, inducing the cysteine to adopt a disallowed backbone conformation^23,24^. In the *S. aureus* GatD crystal structure here reported, the catalytic C94 is located between β4 and α4 (Fig. 2) with a Ramachandran disallowed conformation (Φ = 49.5° and Ψ = −114.0°), similarly to other GATase triads such as CPS^15^ and AS^25^. Moreover, the GatD C94 shows very well-defined electron density. The sequence of the nucleophile elbow motif in *S. aureus* GatD, T92-I93-C94-G95-G96, is not fully conserved in comparison to other triad GATases since the first residue of the G-X-C-X-G motif (glycine) has been replaced by a threonine (Fig. 4a and b). However, the small size of the threonine residue will not hinder the required arrangement of the cysteine within the nucleophile elbow, for the catalytic reaction (Fig. 4c).

### Glutamine sequestration

**Figure 5:**
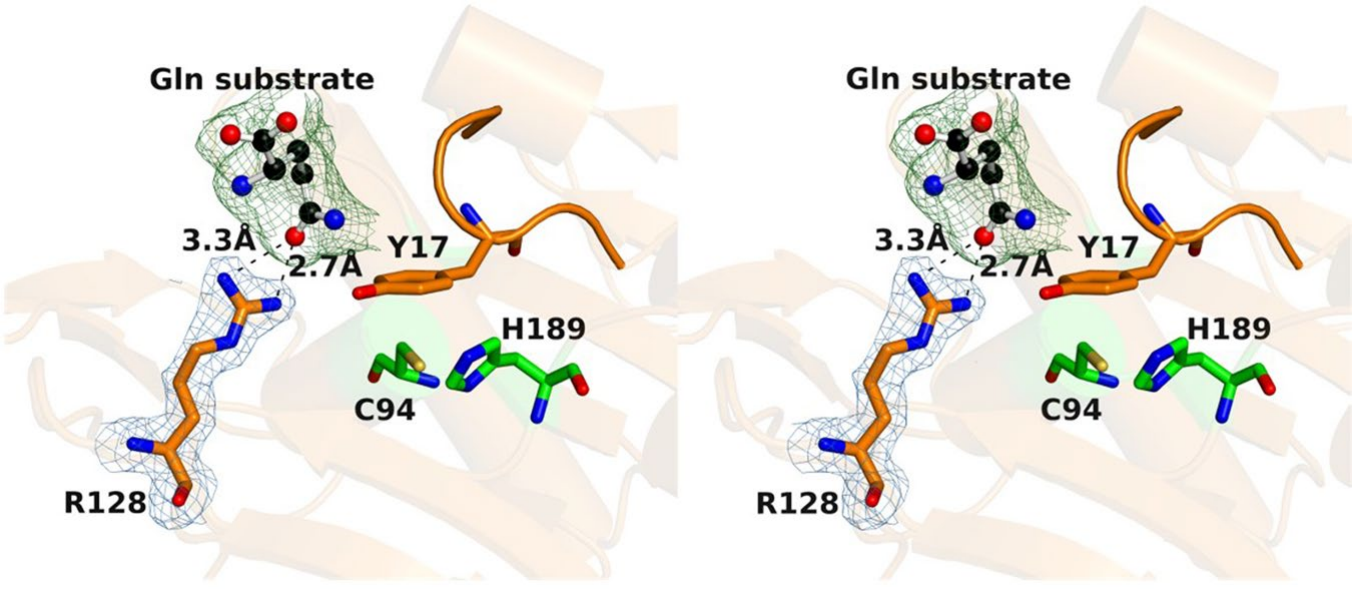
*S. aureus* GatD-glutamine complex in wall-eyed stereo view. The glutamine substrate molecule is hydrogen-bonded to the protein surface residue R128, probably because the loop containing the Y17 amino acid is blocking its entrance to GatD active site. Y17 and R128 residues are represented as sticks with carbon atoms colored orange while glutamine is represented as ball and stick with carbon atoms in black. The catalytic dyad residues C94 and H189 are represented as sticks with carbon atoms colored green. The structure is shown in wall-eyed stereo view and the 2Fo-Fc map for R128 (in blue) contoured at 1.0 σ. Omit map mFo-DFc for glutamine is colored green and contoured at 3.0 σ.

*S. aureus* GatD crystallized in the presence of its substrate, glutamine, which likely arose from the expression conditions of the protein, since it was not added during the purification/crystallization steps. The amino acid molecule is found at the surface of the protein, at 8.6 Å from the nucleophilic thiol group of C94 (Fig. 4c), only interacting with R128 from molecule A. The amide oxygen of the free glutamine is establishing hydrogen bonds with the guanidinium group of R128, with bond distances of 3.3 Å and 2.7 Å (Fig. 5). In this regard, R128 seems to be an important residue for sequestrating and accommodating the substrate at the surface of the protein before directing it to the catalytic site for hydrolysis. It is worth mentioning that, due to crystal packing effects, the glutamine binding site is accessible only in chain A. R128 of chain B is facing chain A, which prevents substrate binding.

Thoden, J.B., *et al*.^26^ reported a crystal structure of the CPS enzyme from *E. coli* (PDB ID 1JDB), in which the glutamine substrate also did not enter the GATase active site, but was located at the surface, near the interface with the synthase domain. In this case, the carboxylic group of the glutamine main chain is hydrogen-bonded to two arginine residues of the glutaminase domain, R120 and R123. Additionally, in the structure of cytidine triphosphate synthetase from *Thermus thermophilus* (PDB ID 1VCO), the enzyme was crystallized in the presence of glutamine, which was found at the active site^19^. The authors claim that a hydrogen bond between the amine group of R472 main chain and the α-carboxylate group of glutamine is potentially important for substrate stabilization at the active site. Together, these results suggest that the presence of arginine residues can play an important role in glutamine sequestration for subsequent hydrolysis.

According to the related structures described in the literature, acceptor binding or complex assembly promote the reorganization of a loop at the surface of the glutaminase domain that allows glutamine entrance^13^. This loop may shield the glutaminase active site, preventing the deamidation reaction in the absence of the synthase domain. In *S. aureus* GatD, this corresponds to residues _14_LNLYSD_19_, which are flanked by helices α1 and α2. The GatD-glutamine complex here reported shows the Y17 side chain nearly co-planar to the H189 imidazole ring and establishing a hydrophobic interaction with C94 (Fig. 5). In this conformation, Y17 is obstructing the access of the free glutamine to the active site.

### Structural insights into the *S. aureus* GatD sequence homologs

GatD has been assigned as belonging to the group of prokaryotic enzymes involved in cobalamin biosynthesis, as cobyrinic acid a,c-diamide synthase (CobB) or cobyric acid synthase (CobQ)^9^. CobB^27^ and CobQ^28^ have been mostly studied in *Pseudomonas denitrificans*. CobB has glutaminase activity, catalyzing the conversion of cobyrinic acid to cobyrinic acid a,c-diamide^27^, while CobQ is responsible for the amidation reaction of 5′-deoxy-5′-adenosyl-cobyrinic acid a,c-diamide to cobyric acid^28^. Both proteins are organized in two domains, the N-terminal synthase domain with an ATP-binding motif, and a glutaminase domain at the C-terminal region.

Using *S. aureus* GatD sequence as query in a BLASTP search retrieves a set of targets, all belonging to the CobQ family of proteins, with higher homology towards a probable cobyric acid synthase protein from *Methanocella arvoryzae* (Table 2). Differentiation between CobQ and CobB proteins is not clear since their functional and structural information is scarce and different views about domain organization have been discussed, namely including them in the class I triad GATase^24^ or in a, so far, non-classified group of GATases^12^. Sequence alignment of CobB and CobQ proteins with triad GATases shows that only the catalytic cysteine and histidine residues are conserved^29^. The lack of the catalytic glutamate suggests that these enzymes can perform the glutaminase activity as functional dyads, as *S. aureus* GatD, or might have other residue to perform the role of glutamate in a potential triad^29^.

**Table 2:** Top 10 Unique Hits from BLASTP Analysis of GatD Sequence.

Protein	Organism	Blast Score	Protein length	Identity	E_value	Uniprot
Probable cobyric acid synthase	*Methanocella arvoryzae*	63.5	487	202 (30%)	1.81^−10^	Q0W1N4
Cobyric acid synthase	*Listeria monocytogenes*	62.8	511	188 (30%)	3.46^−10^	Q8Y7R3
Cobyric acid synthase	*Listeria innocua*	60.8	511	155 (32%)	1.36^−09^	Q92CK0
Probable cobyric acid synthase	*Methanosphaerula palustris*	60.1	487	191 (30%)	2.44^−09^	B8GDE3
Cobyric acid synthase	*Listeria welshimeri*	60.1	511	153 (31%)	3.12^−09^	A0AHV1
Probable cobyric acid synthase	*Methanococcus maripaludis*	57.4	492	158 (34%)	1.86^−08^	A4FWW2
Cobyric acid synthase	*Desulfitobacterium hafniense*	57.4	514	142 (32%)	2.32^−08^	Q24Q41
Probable cobyric acid synthase	*Methanopyrus kandleri*	56.6	494	125 (34%)	4.24^−08^	Q8TVH5
Cobyric acid synthase	*Bacteroides fragilis*	55.8	495	187 (26%)	6.09^−08^	Q64TD9
Probable cobyric acid synthase	*Methanothermobacter thermautotrophicus*	55.5	504	163 (32%)	9.04^−08^	O26880

^a^Identity includes the number of aligned residues with *S. aureus* GatD sequence and the percentage of identical residues.

As expected, *S. aureus* GatD only aligns with the C-terminal domain of Cob proteins, which corresponds to the glutaminase domain of these enzymes (Supplementary Fig. S3). Based on this sequence alignment, the important features in the GatD crystal structure here described seem to be conserved in CobB/CobQ proteins, mainly in CobQ. In addition to the catalytic dyad, the residues next to C94 and H189 (using GatD numbering) are conserved (Supplementary Fig. S3), suggesting that the architecture of the active site of both CobQ and GatD are probably very similar. High sequence homology is also observed for the oxyanion hole and the nucleophile elbow (although, for CobQ proteins, the first residue of the motif is a glycine while in GatD it is a threonine). Interestingly, R128 of GatD aligns in most cases with positively charged lysine residues that might be responsible for substrate interaction prior to binding at the glutaminase active site. Both CobB and CobQ proteins show the presence of extra C-terminal amino acids that match the extra α-helix of GatD (Supplementary Fig. S3) but are absent in all other GATases characterized so far (Supplementary Fig. S2). According to secondary structure prediction software^30^, these residues can also form an α-helix that will likely superimpose with the C-terminal region of GatD. Grounded on all these structural evidences, we suggest that the *S. aureus* GatD crystal structure might be the first representative of a third class of GATases, where CobQ and CobB enzymes are included.

### Comparison between the *S. aureus* GatD and pathogenic Gram-positive homologs

**Figure 6:**
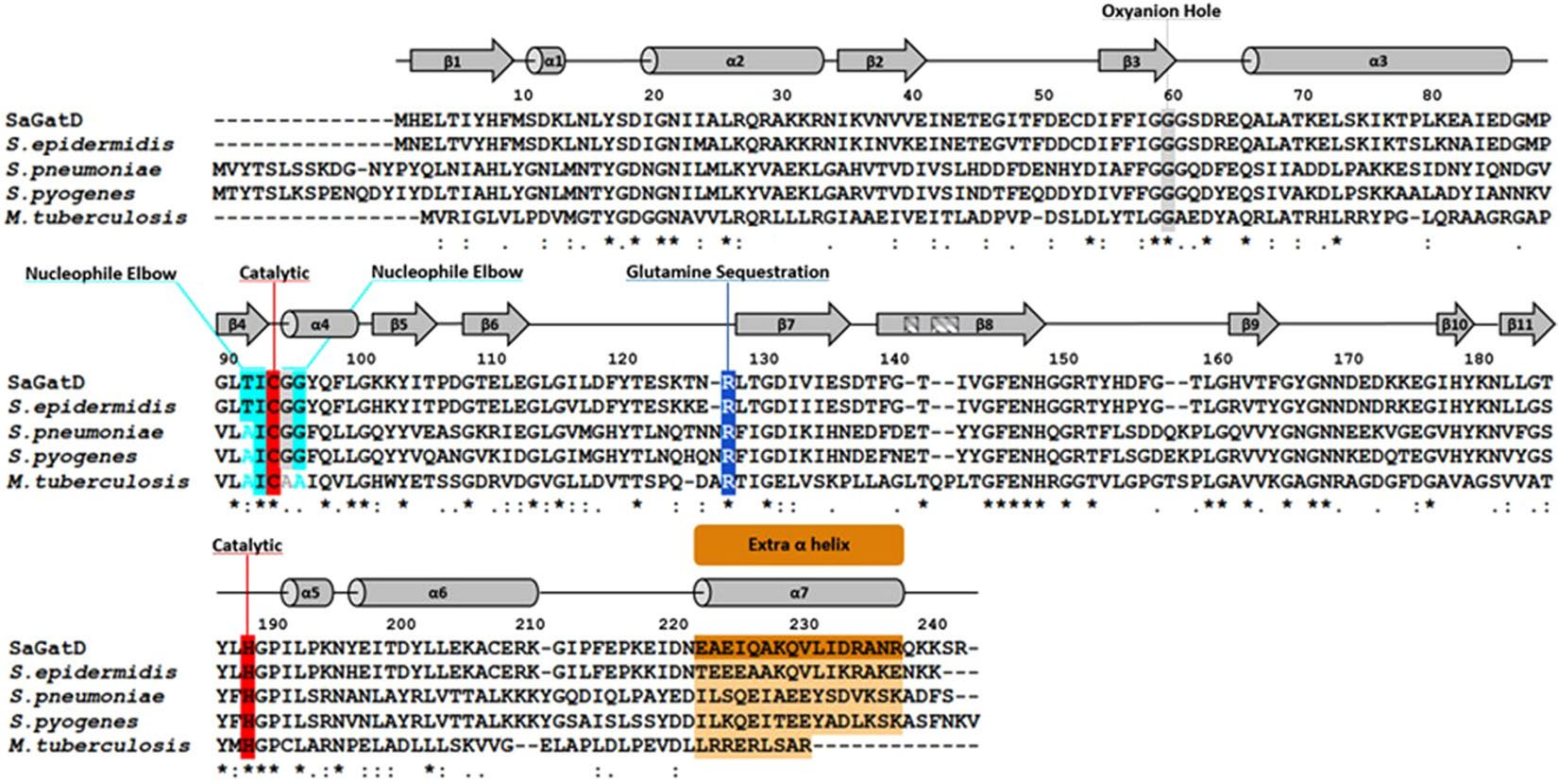
Multiple sequence alignment of GatD proteins from pathogenic Gram-positive bacteria. *S. aureus* GatD (represented in the alignment as SaGatD) was aligned using the ClustalW algorithm with the amino acid sequences of GatD proteins from *S. epidermidis*, *S. pneumonia*, *S. pyogenes* and *M. tuberculosis* (with UNIPROT codes A0A0H2VH11, Q8DNZ8, Q1JH53 and I6XI14, respectively). Important GatD residues are highlighted such as the catalytic C94 and H189 dyad in red, oxyanion hole G60 and G95 in gray, nucleophile elbow T92, I93 and G96 in cyan, glutamine sequestration R128 in blue and the extra C-terminal α-helix in orange. The secondary structural elements are shown above the aligned sequences with α-helices represented as cylinders and β-sheets as arrows.

Given the importance of PG amidation in cell viability, the *S. aureus* GatD structure can be used to derive valuable information for the MurT-GatD homologous systems in other pathogenic Gram-positive bacteria. Protein sequence alignment of *S. aureus* GatD and its homologs from *Staphylococcus epidermidis*, *Mycobacterium tuberculosis*, *S. pneumoniae* and *Streptococcus pyogenes* reveals, as expected, high homology with sequence identity of 86%, 36%, 40% and 43%, respectively (Fig. 6). The catalytic cysteine and histidine of *S. aureus* GatD are conserved in all the aligned proteins, for which the putative triad glutamate residue is also missing. The oxyanion hole and the nucleophile elbow are also highly conserved, suggesting that the architecture of the *S. aureus* GatD active site is preserved. Interestingly, *S. aureus* GatD R128 and extra C-terminal residues are conserved in all sequences. These results indicate that Gram-positive GatD proteins diverge from the well-known triad GATases. The conservation of the loop capping the active site in these systems, especially Y17, makes this region a good target for inhibition strategies, imprisoning the enzyme in a nonproductive conformation.

### *In vitro* glutaminase activity of *S. aureus* MurT-GatD

**Figure 7:**
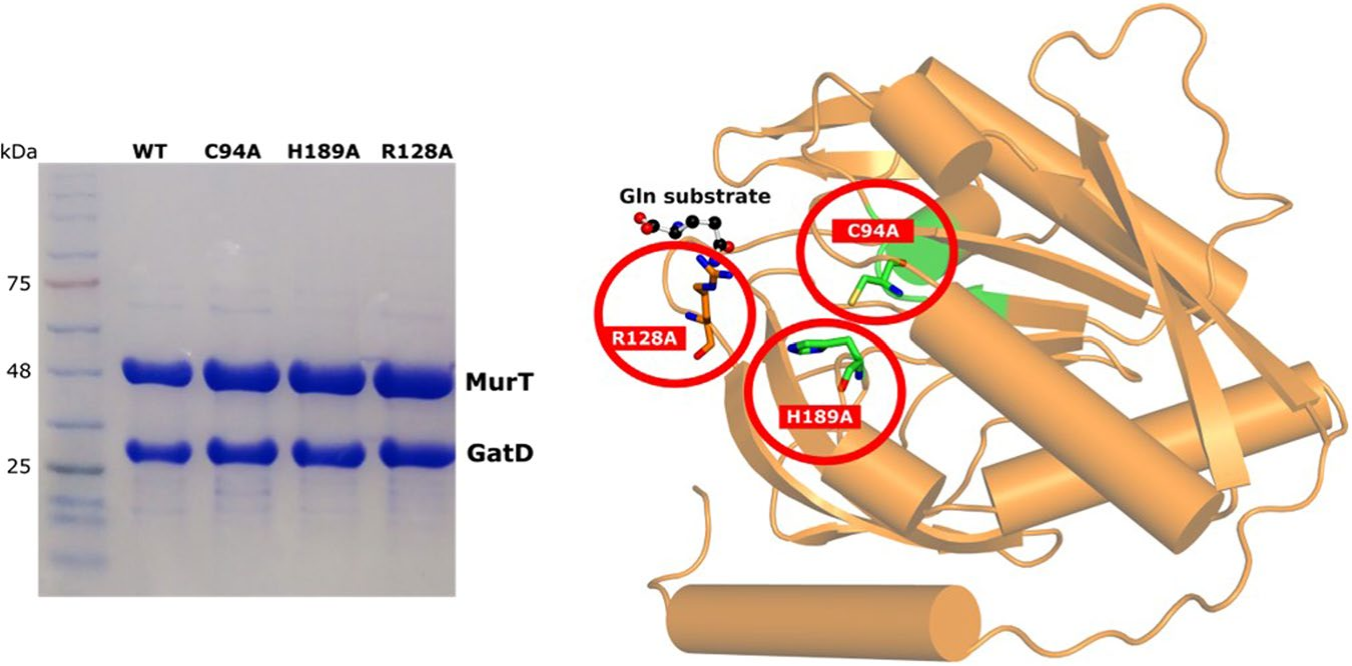
Purity of the heteromeric complex MurT-GatD. 12% SDS-PAGE gel of the final samples of *S. aureus* MurT-GatD *wt* and C94A, H189A and R128A mutants prior to enzymatic activity tests. Insert, *S. aureus* GatD cartoon highlighting the mutated residues that were prepared for further functional studies.

The analysis of GatD crystallographic data provides strong evidence for the involvement of R128 in the glutamine sequestration, together with some important residues in the catalytic dyad, such as C94 and H189. To further validate this hypothesis, the cloning construct containing *murT* and *gatD* genes was modified to introduce point-mutations, replacing the highlighted amino acids by alanines in GatD protein. The resulting protein complexes MurT-GatD *wt* and mutants C94A, H189A, R128A were produced and their glutaminase activity evaluated using ^1^H-NMR spectroscopy. Expression and purification of *S. aureus* MurT-GatD *wt* and the selected mutants led to similar yields of pure and homogeneous MurT-GatD protein complexes. The final samples showed a molar ratio of MurT-GatD of 1:1 (Fig. 7), confirming the heteromeric complex formation in solution, suggested by Münch, D., *et al*.^8^. The stability of the produced MurT-GatD variants show that the mutations imposed in GatD protein did not affect complex formation or solubility.

**Figure 8:**
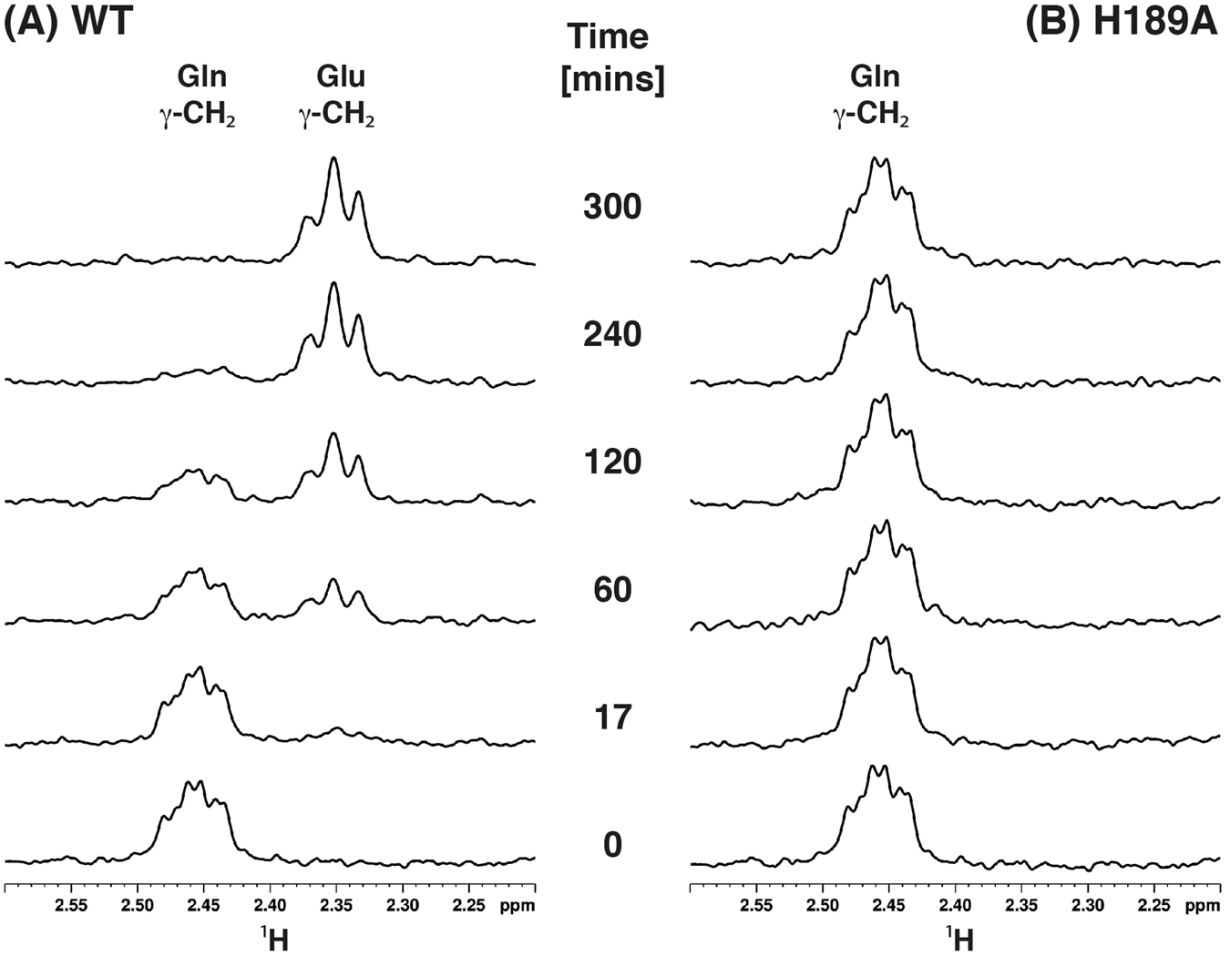
Glutaminase activity monitored by ^1^H-NMR. Expansions of the ^1^H-NMR spectra of the Gln/Glu γ-CH_2_ peak region with resonance assignment for different reaction times to monitor glutaminase activity for (**A**) *wt* and (**B**) H189A. Initial samples were 875 µM Gln with 35 µM MGH.

**Figure 9:**
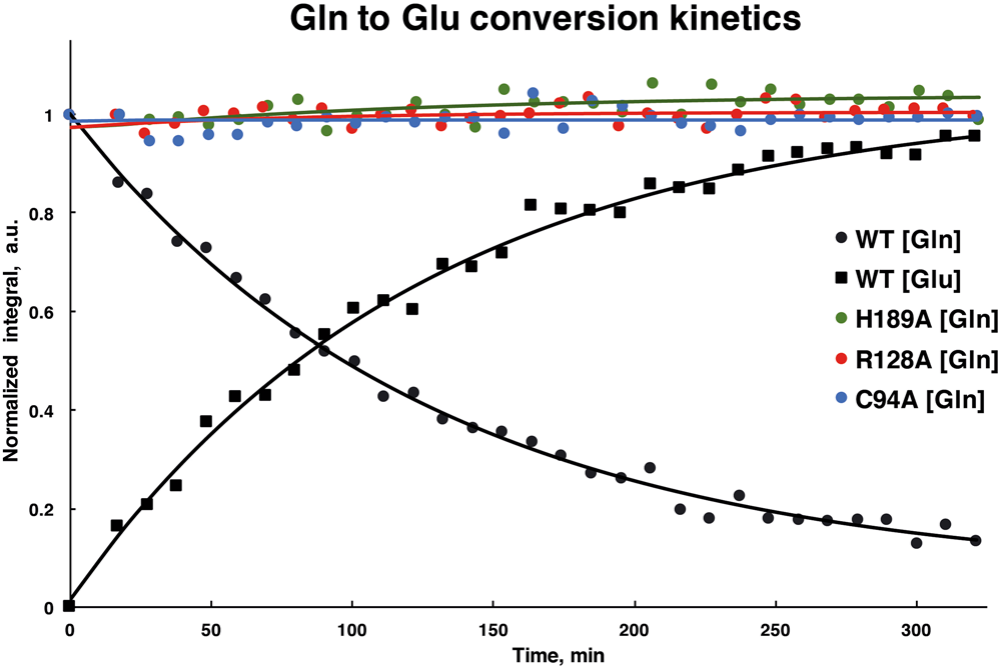
Glutaminase activity kinetics of MurT-GatD protein complexes. Kinetics of glutamine conversion into glutamate was determined from the integration of their γ-CH_2_ peaks for MurT-GatD *wt* and mutants C94A, H189A, R128A. Peak intensities were normalized to the initial concentration of glutamine at t = 0. The error estimated from signal to noise ratio was 4.3% for all experiments. The curves for MurT-GatD *wt* were obtained by fitting the data to a first order rate law but the curves for the mutants are just guidelines for better visualization and comparison.

Glutaminase activity of *S. aureus* MurT-GatD *wt* and variants was followed by ^1^H-NMR spectroscopy (Supplementary Fig. S4). The NMR based activity assay is possible because glutamine and glutamate can be easily distinguished in the ^1^H-NMR spectrum, allowing to follow and quantify the glutamine conversion into glutamate simply by recording and analyzing ^1^H-NMR spectra over time (spectra of MurT-GatD *wt* and H189A mutant in Fig. 8 and C94A and R128A spectra in Supplementary Figure S5). The clearly resolved resonances of glutamine and glutamate γ-CH_2_ protons at 2.458 ppm and 2.348 ppm, respectively, were integrated to quantify the relative concentration of both amino acids in solution for each time point, as depicted in Fig. 9. These results show that MurT-GatD *wt* can convert glutamine into glutamate with a first order rate constant of k = 0.0080 ± 0.0001 min^−1^. This kinetic constant is in agreement with *T. maritima* IGPS glutaminase kinetics in the absence of the synthase substrate^21^.

The glutaminase activity of the MurT-GatD *wt* complex show that GatD is active in the absence of lipid II, which is the ammonia acceptor and MurT substrate. Since the ^1^H-NMR signals of glutamate can be detected, we conclude that the product of the glutaminase activity is released from the MurT-GatD complex, allowing a new glutamine molecule to be deamidated. During the catalytic cycle, the Y17 loop is likely displaced, promoting glutamine entrance to the GatD active site.

In the presence of lipid II, it is likely that the GatD active site will rearrange to a fully active state. The new position of the catalytic residues may affect the conversion rate of glutamine into glutamate. This hypothesis is supported by the 1000-fold increase in glutaminase kinetics of *T. maritima* IGPS when the synthase substrate is present^21^.

The conversion of glutamine into glutamate could be followed in MurT-GatD *wt* glutaminase reaction, while the concentration of glutamine maintained constant along the reaction time for the mutant forms and no glutamate was detected in solution (Fig. 9). This confirms that GatD mutations in C94, H189 and R128 residues impair completely the glutaminase activity of the MurT-GatD complex. This establishes the selected residues as essential for the glutaminase activity. Similar studies performed on class I triad GATase CPS protein also showed that point mutation in the catalytic cysteine and histidine produced completely inactive protein forms^31,32^. These results reinforce the crystallographic data confirming the catalytic architecture of GatD active site, composed by these two residues. Furthermore, our results show univocally that R128 is determinant for glutaminase activity. The amine-based side chain must be detrimental to capture the substrate and drive it into the active site for catalysis.

## Conclusions

Multidrug resistant Gram-positive bacteria are a major threat to human health, *Staphylococcus aureus* being one of the leading causes of infection worldwide. Many efforts have been made to find new antibiotics and develop new strategies to fight antibiotic resistant bacteria. The enzymatic machinery involved in PG biosynthesis is one of the most common targets for drug development, since it is vital for shape maintenance and turgor pressure counterbalance. The amidation reaction is one of the crucial steps of this biosynthetic process and is achieved by the bi-enzymatic complex MurT-GatD. GatD is responsible for metabolizing glutamine and providing ammonia to MurT, the ligase that converts D-glutamate into D-iso-glutamine. At the sequence level, *S. aureus* GatD is similar to other pathogenic Gram-positive bacteria and to CobB and CobQ-related enzymes, suggesting that these enzymes can be grouped in a new sub-family of GATase domains. The high-resolution crystal structure of *S. aureus* GatD showed unique features when compared to other GATase domain architectures. Complementary MurT-GatD glutaminase activity results revealed that GatD residues C94 and H189 are the catalytic amino acids of the active site. This arrangement suggests that GatD may rely on a catalytic dyad although, we cannot exclude putative conformational changes upon MurT/lipid II binding that may bring a third residue to participate in the enzymatic reaction. Glutamine sequestration by GatD is mediated by R128, while Y17 seems to block glutamine entrance, in the absence of MurT and lipid II. MurT binding and full complex formation seem to induce a GatD conformational change, adopting its active structural configuration. Even in the absence of lipid II, the MurT-GatD complex has glutaminase activity.

The structural features of *S. aureus* GatD identified in this work are promising hints for the development of small molecules capable to impair lipid II amidation, placing MurT-GatD as a potential new drug target to fight multidrug resistant bacteria.

## Methods

### GatD expression, purification and crystallization

*S. aureus* GatD was cloned, expressed, purified and crystallized as previously described^33^. Briefly, the PCR amplified sequence of GatD was cloned into the pOPINF vector (Supplementary Table 1) using the In-Fusion method^34^. The protein was expressed by the auto-induction method^35^ in *E.coli* strain Lemo21(DE3) and purified in two chromatographic steps: IMAC and SEC. The crystallization screens were performed at a high-throughput facility (OPPF-UK) using a Cartesian instrument^33^. Native and Se-Met labeled protein crystals were obtained by vapor diffusion using polyethylene glycol (PEG) as precipitating agent. The procedure from protein expression to crystallization is described in^33^.

### Data collection ans structure determination

X-ray diffraction data of native GatD crystals were collected at temperature 100 K at beamline I02 (λ = 1.0000 Å), Diamond Light Source, UK. GatD crystallized in the space group P2_1_2_1_2_1_ with unit cell dimensions of *a* = 48.61 Å, *b* = 93.92 Å, *c* = 110.08 Å, containing 2 molecules per asymmetric unit and a solvent content of 47%. The data was processed using Xia2^36,37^ through the automatic software pipeline available at Diamond Light Source. The phase problem was solved by SAD using a Se-Met derivative, as described in^33^. The experimental phases were later used to solve the structure of GatD in the native dataset (at 1.85 Å resolution). The structure was manually built using Coot^38^ and refined using Phenix, considering restraint refinement with 15 TLS groups and isotropic B-factors^39^. After several rounds of manual rebuilding and refinement the R and R_free_ of the final model converged to 0.149 and 0.186. To avoid bulk solvent mask effect on the modeled glutamine molecule, an omit mFo-DFc difference map was generated for the glutamine using phenix. polder program^40^, where the glutamine region within a radius of 10 Å was excluded (Supplementary Figure S6).

Automatic water picking followed by manual examination allowed identifying 625 water molecules, one PEG molecule, and one glutamine molecule. The structure quality was validated using MolProbity^41^. Data collection and refinement statistics are summarized in Table 3. Atomic coordinates and structure factors have been deposited in the Protein Data Bank (PDB) with the accession code 5N9M.

**Table 3:** Data collection and refinement statistics.

	GatD-glutamine complex
**Data collection**	
Space group	P2_1_2_1_2_1_
**Cell dimensions**	
*a*, *b*, *c* (Å)	48.61, 93.92, 110.08
α, β, γ (°)	90, 90, 90
Resolution (Å)	36.44–1.85 (1.89-1.85)^*^
*R*_pim_	0.028 (0.313)
*I*/σ*I*	25.4 (5.2)
Completeness (%)	100.0 (99.9)
Redundancy	21.7 (20.1)
**Refinement**	
Resolution (Å)	36.44–1.85
No. reflections	42922
*R*_work_/*R*_free_	0.149**/**0.186
**No. atoms**	
Protein	3893
Ligand/ion	21
Water	625
***B*** **-factors**	
Protein	27.43
Ligand/ion	43.47
Water	33.57
**R.m.s. deviations**	
Bond lengths (Å)	0.006
Bond angles (°)	0.990
**Protein geometry**	
Poor rotamers	1 (0.24%)
Ramachandran outliers	0 (0.00%)
Ramachandran favored	467 (97.9%)
Cβ deviations >0.25 Å	0 (0.00%)
Bad backbone bonds	0**/**3980 (0.00%)
Bad backbone angles	0**/**5369 (0.00%)

The dataset was collected from a single crystal. ^*^Highest resolution shell is shown in parenthesis.

### Sequence homology studies

The BLAST search (https://blast.ncbi.nlm.nih.gov/Blast.cgi) was achieved using GatD sequence as query against UniProtKB/Swiss-Prot database in the standard protein blast (blastp). All multiple sequence alignments were performed using the ClustalW algorithm^42^.

### Cloning and production of *S. aureus* MurT-GatD *wt* and mutants

The coding regions for *S. aureus murT-gatD* genes were amplified by PCR from total DNA obtained from *S. aureus* strain COL (Supplementary Table 1) and the flanking restriction sites *Nco I* and *Xho I* were introduced in the resulting DNA fragments by PCR primers. The amplified *murT-gatD* operon was cloned into the *Nco I – Xho I* sites of the expression plasmid pET28a (Supplementary Table 1). The resulting plasmid pET28a-*murT-gatD*-His_6_ was introduced into *E. coli* (DE3) CodonPlus RIPL (Stratagene).

Site-directed mutagenesis was used to produce three variants of MurT-GatD *wt*, where C94, H189 and R128 from GatD protein were altered to alanine. The base pair exchanges were introduced in *gatD* gene on plasmid pET28a-*murT-gatD*-His_6_ by PCR mutagenesis using Phusion DNA Polymerase (Thermo Fisher Scientific) resulting in the plasmids pET28a-*murT-gatD*-His_6_C94A, pET28a-*murT-gatD*-His_6_H189A and pET28a-*murT-gatD*-His_6_R128A (Supplementary Table 1). All primers are shown in Supplementary Table 2. Presence of the mutations was confirmed by sequencing.

### *S. aureus* MurT-GatD *wt* and mutants expression and purification

*Escherichia coli* BL21-CodonPlus (DE3)-RIPL cells (Agilent) were used for heterologous expression of *S. aureus* MurT-GatD *wt* and mutants C94A, H189A and R128A, after transformation with plasmids pET28a-*murT-gatD*-His_6_C94A, pET28a-*murT-gatD*-His_6_H189A and pET28a-*murT-gatD*-His_6_R128A, respectively. Cells were grown aerobically in LB medium at 310 K and then the media was supplemented with 0.5 mM isopropyl β-D-1-thiogalactopyranoside to induce protein expression at 293 K and 150 rpm for 16 hours. The cells were harvested by centrifugation at 7500 × *g* for 15 minutes. The cell pellet was resuspended in buffer A (100 mM Tris-HCl, pH 8.2, 500 mM NaCl, 10 mM MgCl_2_, 5 mM 2-mercaptoethanol) supplemented with 10 mM imidazole, 10 μg/mL DNaseI and ½ tablet of cOmplete™ ULTRA Tablets, Mini, EDTA-free, EASYpack Protease Inhibitor Cocktail (Roche). Cells lysis was completed by sonication and the resulting supernatant was applied onto a 5 mL HisTrap column (GE Healthcare). Bound protein was eluted using a linear gradient of imidazole. The fractions, where the presence of the complex MurT-GatD was confirmed by SDS-PAGE, were pooled and concentrated. The resulting sample was applied onto a Superdex 200 10/300 GL (GE Healthcare) pre-equilibrated with buffer A. The peak fractions were pooled and concentrated to a final concentration of 70 µM. Buffer exchange to 50 mM Tris-d11, 500 mM NaCl, 10 mM MgCl_2_, 5 mM 2-mercaptoethanol in D_2_O was performed using a PD MiniTrap G-25 (GE Healthcare).

### NMR experiments

All NMR experiments were performed at 293 K using a Bruker Avance III 400 operating at 400.15 MHz for protons, equipped with a 5 mm BBO probe head. Enzyme kinetics was followed by ^1^H-NMR spectroscopy measuring the conversion of glutamine into glutamate by integration of their γ-CH_2_ resonance over time. In a typical experiment, the MurT-GatD protein sample (*wt*, H189A, R128A or C94A) was added in a 5 mm NMR tube containing a solution of glutamine and ^1^H-NMR spectra were recorded approximately every 12 minutes for 320 minutes. The initial glutamine concentration was 875 µM in a 99.9% D_2_O buffered solution (pH meter reading pH 7.9, uncorrected for deuterium isotope effect, 50 mM Tris-d11, 500 mM NaCl, 10 mM MgCl_2_, 5 mM 2-mercaptoethanol, and 50 µM TSP) and the sample protein concentration was 35 µM (i.e. protein:glutamine ratio of 1:25). ^1^H-NMR spectra were acquired with 16 K data points and 256 scans in a spectral window of 6009.6 Hz centered at the water resonance (1880.5 Hz) with a T_1*ρ*_ relaxation filter of 35 ms to suppress protein signals. Chemical shifts were calibrated relative to TSP as internal reference. NMR data was processed in Bruker TopSpin™ 3.5 and analysed with Bruker Dynamics Center™ 2.5. Errors were determined based on the signal to noise ratio.

## Electronic supplementary material

Supplementary Information
