# Supplementary material for: First insights of peptidoglycan amidation in Gram-positive bacteria - the high-resolution crystal structure of Staphylococcus aureus glutamine amidotransferase GatD
Source: Sci Rep. 2018 Mar 28;8:5313. doi: 10.1038/s41598-018-22986-3 (PMC5871853; doi:10.1038/s41598-018-22986-3)
Supplement: Supplementary file 1 — Supplementary Information [file 41598_2018_22986_MOESM1_ESM.pdf]

First insights of peptidoglycan amidation in Gram-positive bacteria - the high-resolution crystal structure of *Staphylococcus aureus* glutamine amidotransferase GatD

Francisco Leisico[1¶], Diana Vieira[1,2¶], Teresa A. Figueiredo[1,3], Micael Silva[1], Eurico J. Cabrita[1] Rita G. Sobral[1], Ana Madalena Ludovice[1], José Trincão[4], Maria João Romão[1], Hermínia de Lencastre[3,5*] and Teresa Santos-Silva[1*]

Email: tsss@fct.unl.pt and lencash@mail.rockefeller.edu

¶These authors contributed equally to this work.

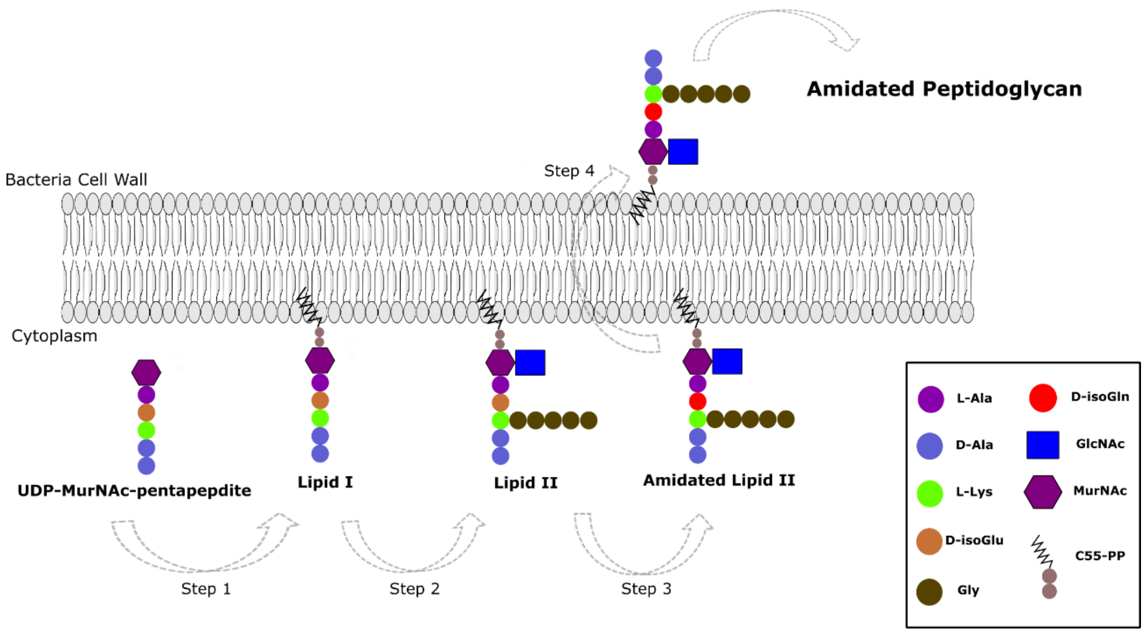

Supplementary Figure S1. General schematic representation of *S. aureus* lipid II biosynthesis.

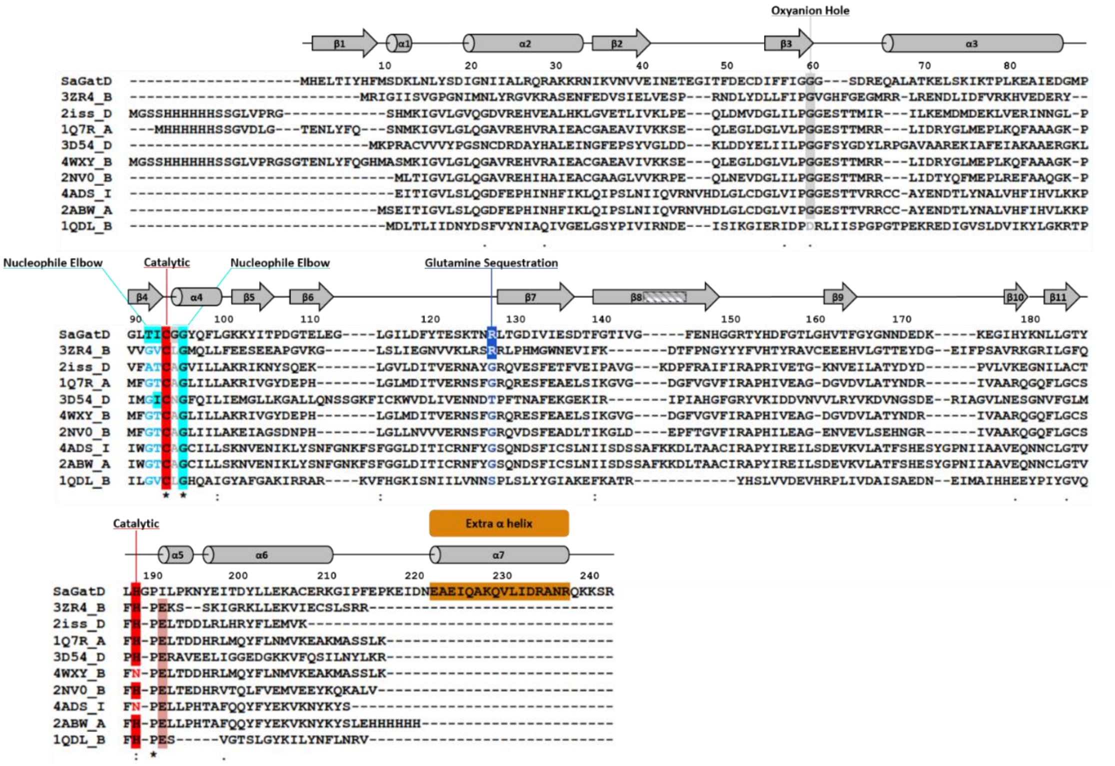

Supplementary Figure S2. Multiple sequence alignment of *S. aureus* GatD and PDBeFold target sequences. *S. aureus* GatD amino acid sequence (represented in the alignment as SaGatD) was aligned using the ClustalW algorithm with the amino acid sequences of PDBeFold target proteins presented in Table 1 (here represented with the respective PDB ID and chain). Important GatD residues (and the respective aligned amino acids) are highlighted: the potential catalytic dyad C94 and H189 in red, oxyanion hole G60 and G95 in gray, nucleophile elbow T92, I93 and G96 in cyan, glutamine sequestration R128 in blue and the extra C-terminal α helix of GatD in orange. The secondary structural elements from the *S. aureus* GatD structure are shown above the aligned sequences with α-helices represented as cylinders and β-sheets as arrows. The glutamate residue belonging to the catalytic triad of the identified PDBeFold targets are highlighted in salmon.

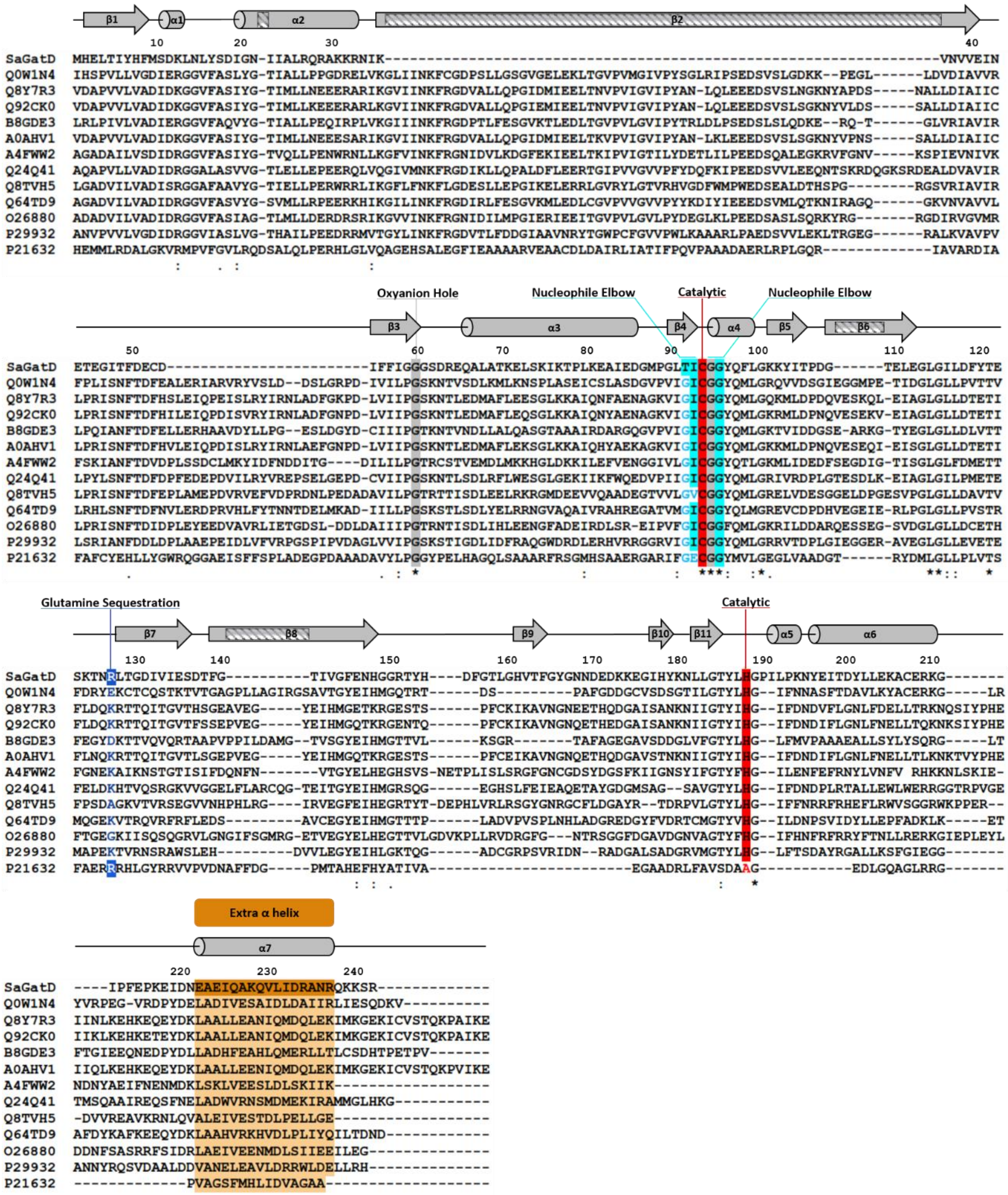

Supplementary Figure S3. Multiple sequence alignment of *S. aureus* GatD and homologs.

S. aureus GatD (represented in the alignment as SaGatD) was aligned using the ClustalW algorithm with the amino acid sequences of the BLASTP hits (indicated in the alignment with the respective UNIPROT code) and also with CobQ and CobB from *P. denitrificans*, (UNIPROT code P29932 and P21632, respectively). Important GatD residues are highlighted such as the catalytic dyad C94 and H189 in red, oxyanion hole G60 and G95 in gray, nucleophile elbow T92, I93 and G96 in cyan, glutamine sequestration R128 in blue and the extra C-terminal α helix of GatD in orange. The secondary structural elements are shown above the aligned sequences with α-helices represented as cylinders and β-sheets as arrows.

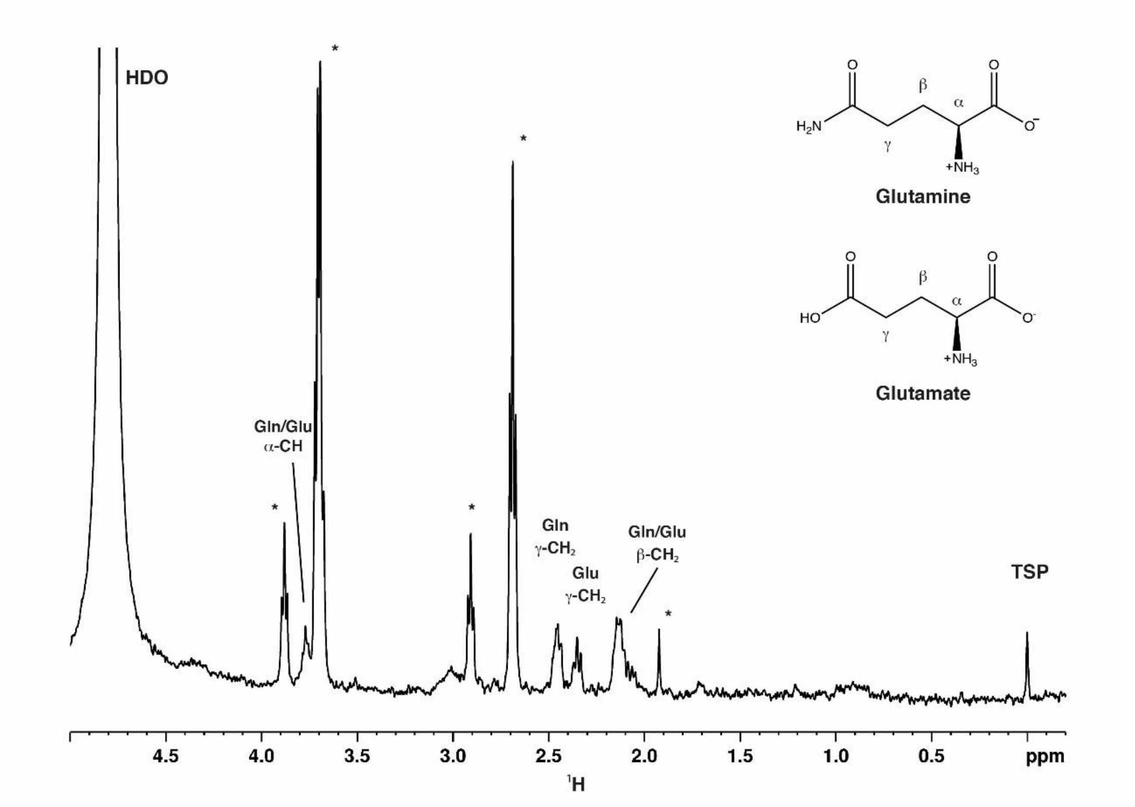

Supplementary Figure S4. Representative ^{1}H-NMR-based glutaminase activity of MurT-GatD *wt*.

Resonance assignments show the distinct chemical shift of the γ-CH_2 of glutamine and glutamate resonances that allow the quantification of protein activity. The ^{1}H-NMR spectrum here presented shows glutamine (875 µM) conversion in glutamate catalyzed by 35 µM of MurT-GatD *wt* after 60 minutes of reaction. Asterisk (*) indicates buffer resonances.

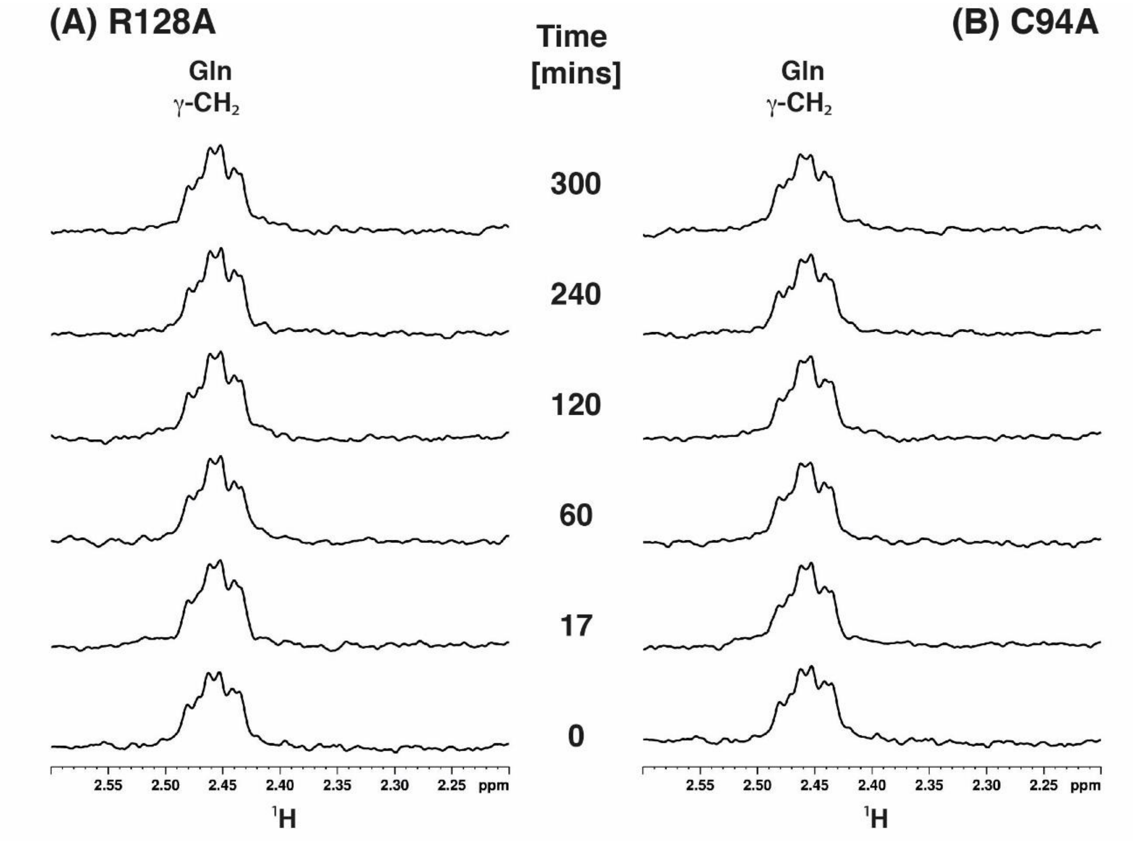

Supplementary Figure S5. Glutaminase activity of MurT-GatD mutants monitored by ^{1}H-NMR.

Expansions of the ^{1}H-NMR spectra of the glutamine and glutamate γ-CH_2 peak region with resonance assignment for different reaction times to monitor glutaminase activity for (A) R128A and (B) C94A mutants. The reactions were performed with 875 µM of glutamine and 35 µM of protein. Glutamine concentration remains constant over time in (A) and (B) and no glutamate formation can be detected.

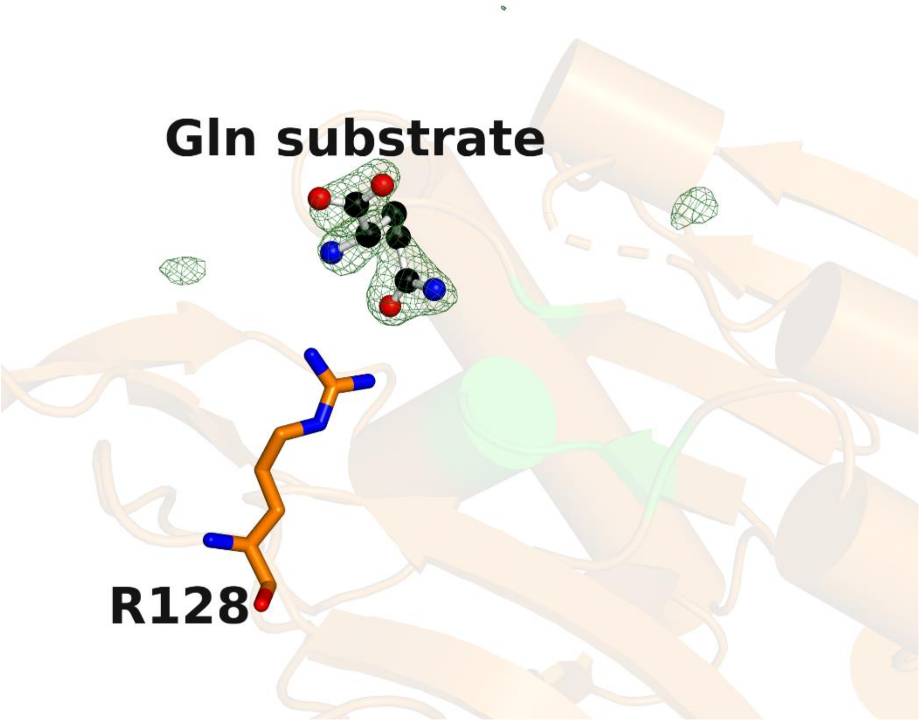

Supplementary Figure S6. Confirmation of glutamine substrate presence by polder map.

Polder map was generated excluding the bulk solvent contribution within a 10 Å radius from glutamine substrate. The resulting polder map is contoured at 5 σ and is presented within a 10 Å radius from glutamine substrate to show that there are no other residual density peaks besides glutamine.

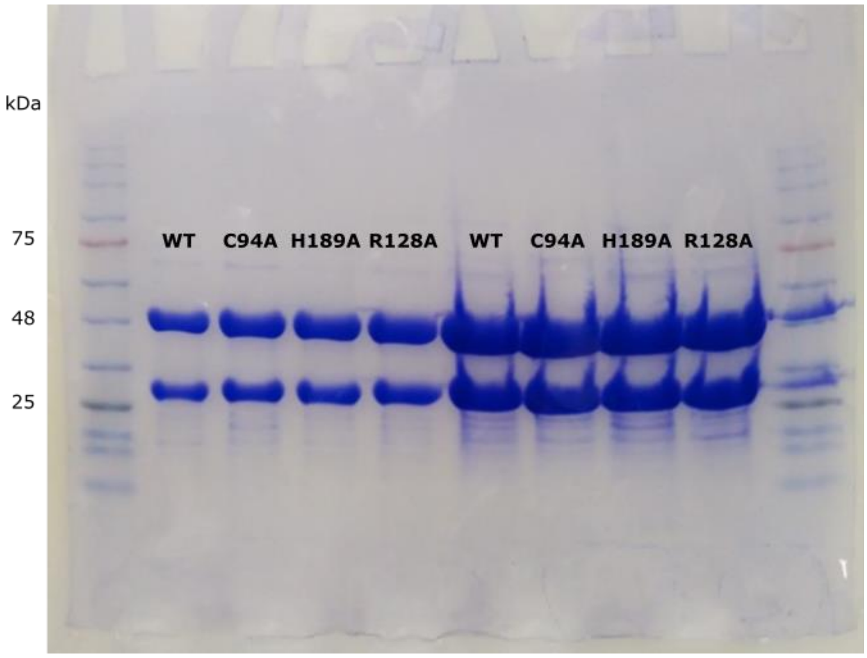

Supplementary Figure S7. Full-length SDS-PAGE gel used to assess the heteromeric complex MurT-GatD purity.

Full-length 12% SDS-PAGE gel of the final samples of *S. aureus* MurT-GatD wt and C94A, H189A and R128A mutants prior to enzymatic activity tests. Two different amounts of protein were loaded as quality check. Both MurT (49 KDa) and GatD (27 KDa) proteins are resolved in the gel.

Supplementary Table S1. Strains and plasmids used in this study.

**Strains**		
S. aureus		
COL	Homogeneous Mc^r	The Rockefeller Univ. Collection
E.coli		
BL21-CodonPlus(DE3)-RIPL	*E. coli* B F^- *ompT hsdS*(r_B^- m_B^-) *dcm*$^+$ Tet^r *gal λ* (DE3) *endA* Hte [*argU proL*Cm^r] [*argU ileY leuW* Strep/$Spec^r$]	Stratagene.
BL21-His_6-*gatD*	BL21-CodonPlus(DE3)-RIPL strain with pET28a-His_6-*gatD*	
BL21-*murT*-*gatD*-His_6	BL21-CodonPlus(DE3)-RIPL strain with pET28a-*murT*-*gatD*-His_6	Figueiredo, T. A. *et al.* [10]
BL21-*murT*-*gatD*-His_6C94A	BL21-*murT*-*gatD*-His_6 with C94A mutation	In this study
BL21-*murT*-*gatD*-His_6H189A	BL21-*murT*-*gatD*-His_6 with H189A mutation	In this study
BL21-*murT*-*gatD*-His_6R128A	BL21-*murT*-*gatD*-His_6 with R128A mutation	In this study
**Plasmids**		
pOPINF	using the In-Fusion™	In this study
pET28a	Expression vector with T7/*lac* promoter, N-terminal His tag, thrombin cleavage site, C-terminal His tag; kan^r	Invitrogen
pET28a-His_6-*gatD*	pET28a expressing His_6-*gatD*	This study
pET28a-*murT*-*gatD*-His_6	pET28a expressing *murT*-*gatD*-His_6	Figueiredo, T. A. *et al.* [10]
pET28a*murT*-*gatD*-His_6C94A	pET28a expressing *murT*-*gatD*-His_6C94A	In this study
pET28a*murTgatD*His_6H189A	pET28a expressing *murT*-*gatD*-His_6H189A	In this study
pET28a*murTgatD*His_6R128A	pET28a expressing *murT*-*gatD*-His_6R128A	In this study

[a] Mc^r methicillin resistant; Cm^r, chloramphenicol resistant; $Spec^r$, spectinomycin resistant; Kan^r, kanamycin resistant.

Supplementary Table S2. Primers for *gatD* gene mutation in *S. aureus murT-gatD* operon.

Mutation	Forward primer (5'- 3' sequence)	Reverse primer (5'- 3' sequence)
C94A	GGTATGCCGGGATTAACGATT**GCT**GGAGGC TATCAATTTTTAGGG	CCCTAAAAATTGATAGCCTCC**AGC**AATCGTTA ATCCCGGCATACC
H189A	GGCATTCATTATAAAAATTTATTAGGTACTTAT TTA**GCT**GGACCAATTTTACCTAAAAATTACG	CGTAATTTTTAGGTAAAATTGGTCC**AGC**TAAA TAAGTACCTAATAAATTTTTATAATGAATGCC
R128A	CTGAATCAAAGACAAAC**GCA**TTAACAGGAGAT ATTGTTATCG	CGATAACAATATCTCCTGTTAA**TGC**GTTTGTC TTTGATTCAG
